# Efficacy and toxicity analysis of ganciclovir in patients with cytomegalovirus lung infection: what is new for target range of therapeutic drug monitoring

**DOI:** 10.1128/spectrum.00461-25

**Published:** 2025-07-08

**Authors:** Dongjie Guo, Xiaoxing Wang, Pengmei Li, Yongguang Shang, Lei Zhang, Yue Chen, Xudong Kong, Wenqian Chen

**Affiliations:** 1 Department of pharmacy, China-Japan Friendship Hospital 36635, Beijing, China; University of Wisconsin-Madison, Madison, Wisconsin, USA

**Keywords:** ganciclovir, cytomegalovirus, lung infection, therapeutic drug monitoring, trough concentration, clinical efficacy, toxicity

## Abstract

To investigate the relationship between ganciclovir trough concentration and its clinical efficacy and safety in patients with pulmonary cytomegalovirus (CMV) infection and to evaluate the target value of therapeutic drug monitoring (TDM) for predicting clinical efficacy or toxicity, an observational study was conducted on patients with CMV lung infection between Jan. 2021 and Dec. 2023. Target trough ganciclovir concentrations were collected during TDM, and clinical outcomes and adverse events (AEs) were assessed. Multiple regression analyses or binary logistic regression were used to analyze the factors affecting ganciclovir trough concentrations and clinical efficacy. The receiver operating characteristic (ROC) curve analysis identified ganciclovir trough concentration cutoffs as predictors of toxicity. Of 149 included patients, only 19.22% of trough concentrations reached the target range of 2–4 μg/mL. Diabetes, organ transplantation, and elevated hemoglobin levels decrease trough concentrations, whereas elevated blood creatinine and continuous renal replacement therapy (CRRT) increase trough concentrations. No relationship was found between the trough concentrations and the clinical outcomes. Organ transplantation, duration of ganciclovir administration, and albumin levels affect clinical efficacy. The ROC analysis identified ganciclovir trough concentrations of 0.985 (AUC = 0.600) and 0.995 µg/mL (AUC = 0.701) to be associated with decreased hemoglobin and elevated blood creatinine levels, respectively. No association was observed between ganciclovir trough concentration and clinical efficacy. However, thresholds for ganciclovir trough concentrations causing decreased hemoglobin and increased blood creatinine levels were identified. Renal replacement therapy is more likely to lead to elevated trough concentrations of ganciclovir. These findings suggest the importance of TDM for ganciclovir, and a new target range should be considered.

**IMPORTANCE:**

An observational study investigated the relationship between ganciclovir trough concentrations and clinical efficacy and safety. Only 19.22% of ganciclovir trough concentrations reached the target range of 2.0–4.0 μg/mL. Diabetes, organ transplantation, and elevated hemoglobin decreased trough concentrations, while elevated creatinine and CRRT increased concentrations. No relationship was found between ganciclovir trough concentrations and clinical outcomes in CMV lung infection. Organ transplantation, duration of ganciclovir administration, and albumin levels affected the clinical efficacy of ganciclovir. Critical ganciclovir trough concentrations of 0.985 and 0.995 μg/mL were associated with toxicity. The findings suggest the importance of therapeutic drug monitoring (TDM) with ganciclovir treatment.

## INTRODUCTION

Human cytomegalovirus (HCMV) primarily affects immunocompromised individuals or causes congenital infections, leading to pneumonia (1). Hematopoietic stem cell transplant (HSCT) and solid organ transplant (SOT) recipients, along with HIV patients, are particularly vulnerable to HCMV pneumonia (2). Though rare, it can also occur in immunocompetent individuals (3, 4). A Korean cohort study (3) reported a higher all-cause mortality in non-transplant CMV patients compared to SOT recipients, indicating severe outcomes beyond severely immunocompromised transplant recipients. Intravenous ganciclovir and oral valganciclovir are the main antiviral treatments for CMV (5). In healthy individuals with normal renal function, ganciclovir trough plasma concentrations (Cmin) were 1 µg/mL at 11 h for a 5 mg/kg dose and 0.6 µg/mL at 7 h for a 2.5 mg/kg dose (6). Ganciclovir clearance, 91% renal, is highly dependent on renal function (7). In SOT recipients, ganciclovir clearance correlates with renal function, requiring dosage adjustments based on the glomerular filtration rate (8). The most significant adverse effect is myelosuppression. In marrow transplant recipients, neutropenia (ANC < 1,000/µL) occurs in 41 to 58% of patients treated with ganciclovir (9), while in SOT patients, it is about 10%. In advanced HIV patients, severe neutropenia (ANC < 500/µL) occurs in 34% treated with ganciclovir.

Therapeutic drug monitoring (TDM) can improve drug efficacy and reduce adverse effects. The role of TDM in optimizing ganciclovir remains debated (8), yet several centers have adopted TDM programs (10–12). Ganciclovir’s narrow therapeutic window associates higher concentrations with toxicity (13–16). Adequate treatment is crucial, especially for patients with high viral loads to prevent resistance (17). TDM assists in assessing serum concentrations and guiding dosage adjustments (11). *In vitro* data suggest serum concentrations of 0.26 to 1.28 µg/mL inhibit CMV replication by 50% (18). The University Medical Center Groningen targets Cmin of 2–4 mg/L for therapeutic ganciclovir (11). Most studies on ganciclovir TDM focus on transplant recipients, with limited research on the broader adult population. A recent study (10) on TDM in adult CMV infection found no clear link between drug exposure and clinical efficacy or safety, similar to findings in transplant recipients (19). Another study (20) reported a significant correlation between ganciclovir area under curve (AUC) or Cmin and decreased leukocytes and increased blood creatinine without quantitative analysis. An *in vitro* study (21) showed increased toxicity in lymphoblastoid cells after 14 days of ganciclovir exposure. No studies have proposed pharmacokinetic/pharmacodynamic (PK/PD) targets or identified TDM cutoff values predicting clinical efficacy or toxicity (22).

This study focused on adult patients with pulmonary cytomegalovirus infections. The objectives were to evaluate factors affecting ganciclovir Cmin, examine its association with clinical efficacy and toxicity, and perform exposure-efficacy analyses to establish a TDM target value for predicting clinical outcomes.

## MATERIALS AND METHODS

### Study design and ethics

Patients with CMV pneumonia who underwent ganciclovir TDM from Jan. 2021 to Dec. 2023 at China-Japan Friendship Hospital were retrospectively analyzed. The inclusion criteria were as follows: (i) age ≥16 years; (ii) CMV circulating threshold (CT) is positive in bronchoalveolar lavage (BAL) test; and (iii) ganciclovir IV therapy with routine TDM. The exclusion criteria included (i) ganciclovir Cmin not at a steady state and (ii) incomplete laboratory or efficacy data.

### TDM strategy

Routine TDM testing was performed after at least three regular doses. Ganciclovir Cmin was collected 30 min before the next dose. Plasma ganciclovir concentration was determined using a validated liquid chromatography-tandem mass spectrometry (LC-MS/MS) method. Ganciclovir-d5 internal standard solution (20 µL) was added to 400 µL of plasma, followed by 600 µL of acetonitrile. After shaking and centrifugation, 900 µL of the supernatant was transferred to a new tube, mixed with 400 µL of dichloromethane, and centrifuged again. The supernatant (0.2 µL) was injected into the LC-MS/MS system equipped with a BEH C18 column (50 × 2.1 mm, 1.7 µm, Waters, USA). The mobile phase consisted of 2 mM ammonium acetate and 0.1% formic acid in water and acetonitrile with 0.1% formic acid. In the multiple reaction monitoring mode, ions monitored were m/z 256.0→135.0 for ganciclovir and 261.0→135.0 for ganciclovir-d5. The concentration range was 0.3125–20 µg·mL^−1^, excluding concentrations below 0.3125 µg·mL^−1^.

### Clinical data collection

For all patients, the following data were collected: gender, age, weight, disease, type of transplant, presence of renal replacement therapy (RRT) (continuous renal replacement therapy [CRRT] or intermittent renal replacement therapy [IRRT]), administration route, dose, duration, and the start and stop dates of ganciclovir medication. Laboratory values included CMV CT, ganciclovir Cmin, white blood cell count (WBC), neutrophil count (NEUT), platelet count (PLT), hemoglobin (HGB), total bilirubin (TBIL), alanine aminotransferase (AST), aspartate aminotransferase (ALT), serum creatinine (SCr), and creatinine clearance (CRCL) (calculated using Cockcroft-Gault).

### Target range of Cmin, clinical efficacy, and adverse effects

For the target range of Cmin, our institution recommends a Cmin range of 2.0 to 4.0 µg/mL for therapeutic ganciclovir use (11).

As regards clinical efficacy, the treatment efficacy was defined by an increase or decrease in CMV CT, whereas a decrease indicated treatment failure.

For the adverse reactions, hepatic and renal functions were evaluated using ALT, AST, TBIL, and SCr levels. Increases were calculated by subtracting baseline levels from end-of-dose levels. An increase to twice the upper limit of normal at the end of dosing was deemed an adverse drug reaction. Myelosuppression was assessed through decreases in WBC, NEUT, PLT, and HGB calculated as the difference between end-of-dose and baseline measurements. Adverse drug reactions were defined as a 20% decrease in WBC, 20% decrease in NEUT, 50% decrease in PLT, and 20% decrease in HGB levels.

### Statistical analysis

Numerical variables are summarized with medians and interquartile ranges (IQRs), while categorical variables use frequencies and percentages. Data normality was assessed before the correlation test to choose between parametric (Pearson’s test) or non-parametric (Spearman’s test) analyses. One-way analysis of variance analyzed group differences. Multiple linear or binary logistic regression identified influencing factors. A receiver operating characteristic (ROC) curve analysis was performed by selecting the average Cmin of ganciclovir and calculating the AUC and the 95% confidence interval (CI). Data analysis was conducted using IBM SPSS Statistics 25 (IBM SPSS, Chicago, IL, USA) and GraphPad Prism 8 (GraphPad Software, San Diego, CA, USA). In multivariate analyses, *P* values < 0.05 were deemed statistically significant.

## RESULTS

### Demographic information

Between Jan. 2021 and Dec. 2023, 149 patients (281 concentrations) were enrolled in the study. The median age was 65, with 103 males (69.13%). Among them, 29 were solid-organ transplant recipients (19.46%): 21 lung, seven renal, and one liver transplants. Only one patient had an allogeneic hematopoietic stem cell transplant (0.67%). The median initial CMV CT was 24.23. All patients were treated with intravenous ganciclovir for cytomegalovirus infection, with a median daily dose of 300 mg and 5.17 mg/kg. Patient details are provided in Table 1.

**TABLE 1:** Patient characteristics (*n* = 149)^*c*^

Characteristics	Value^*a*^
Age, years	65 (16–89）
Sex (male, %）	103 (69.13）
Weight, kg	60 (35–100）
Immunodeficiency background	
Malignant tumor	14 (9.40）
Allogeneic stem cell transplantation	1 (0.67）
Solid organ transplant	29 (19.46）
Lung	21 (14.09）
Kidney	7 (4.70）
Liver	1 (0.67）
Cytomegalovirus (CMV)	
Initial BAL CMV replication	149 (100%)
Baseline CMV CT	24.23 (12.78–28.23）
Baseline biochemical data	
White blood cell count, 10^9^/L	9.26 (0.43–37.95）
Neutrophil count, 10^9^/L	7.49 (0.34–32.22）
Platelet count, 10^9^/L	163 (6–599）
Hemoglobin, g/L	85 (40–171）
SCr, μmol/L	70.40 (6.80–426.00）
Creatinine clearance, mL/min^*b*^	76.27 (11.14–651.59）
Days of hospitalization, days	26 (7–369）
Daily dose, mg	300 (50–1,000）
Daily dose corrected by weight, mg/kg	5.17 (0.91–15.38）

^*a*^ Data are presented as frequency (%) unless denoted as mean ± standard deviation or median (IQR).

^*b*^ Calculated using the Cockcroft-Gault equation.

^*c*^ Cytomegalovirus (CMV), circulating threshold (CT), and serum creatinine (SCr).

### Overview of therapeutic drug monitoring

Among the 281 ganciclovir concentrations, the maximum was 11.94 µg/mL; the minimum was 0.07 µg/mL; and the mean ± SD was 1.78 ± 1.93 µg/mL. The concentrations were categorized into three groups: <2, 2–4, and >4 µg/mL. A total of 54 cases (19.22%) were within the target range, with <2 µg/mL comprising the highest percentage (70.82%) (Table 2).

**TABLE 2:** Distribution of cases of Cmin by level

Concentration range (μg/mL)	Cases	Proportion (%)	Concentrations (mean ± SD, μg/mL)
＜2	199	70.82	0.87 ± 0.53
2–4	54	19.22	2.68 ± 0.52
＞4	28	9.96	6.54 ± 2.12

### Relationship between ganciclovir Cmin and patient prognosis

After ganciclovir treatment, 102 patients (68.46%) had negative CMV results, and seven (4.70%) had elevated CMV CT, resulting in a 73.15% overall treatment efficiency. No significant association was found between ganciclovir Cmin and CMV prognosis (*P* = 0.311). When categorizing CMV prognosis into CT lowering, increase, and negativization, the drug Cmin and C/D in the CT lowering group were lower than those in the negativization and CT elevated groups but without a significant difference (*P* = 0.347, *P* = 0.623) (Table 3).

**TABLE 3:** Relationship between ganciclovir concentrations and patient outcomes^*a*^

Outcome	Trough concentrations (μg/mL)	Standardized drug trough concentration (μg/mL/g)
CMV negativization	1.76 ± 1.88	8.41 ± 13.96
CMV circulating threshold increase	1.93 ± 2.16	9.89 ± 17.93
CMV circulating threshold lowering	1.04 ± 0.81	5.79 ± 7.56
F	1.064	0.474
P	0.347	0.623

^*a*^ Cytomegalovirus (CMV).

The binary logistic regression analysis (Table 4) revealed that organ transplantation (odds ratio [OR] 0.247, 95% CI: 0.093–0.654) negatively impacted CMV prognosis, while extended ganciclovir administration (OR 1.095, 95% CI: 1.019–1.176) and higher patient albumin levels (OR 1.101, 95% CI: 1.043–1.161) were associated with better clinical outcomes in CMV.

**TABLE 4:** Binary logistic regression of factors influencing the clinical efficacy of CMV^*b*^

Variable	Regression coefficient	Standard error	Wald χ^2^	Odds ratio (OR)	95% CI (CI) for OR	*P* value
Sex	0.320	0.444	0.521	1.377	0.577–3.285	0.471
Age, years	0.006	0.014	0.183	1.006	0.979–1.034	0.669
Weight, kg	−0.012	0.016	0.521	0.988	0.957–1.021	0.470
Hospital stay, days	−0.008	0.008	0.978	0.992	0.977–1.008	0.323
Diabetes	−0.641	0.395	2.626	0.527	0.243–1.144	0.105
Transplantation	−1.400	0.498	7.906	0.247	0.093–0.654	0.005^*a*^
Daily dose, mg	−0.001	0.001	1.900	0.999	0.997–1.000	0.168
Duration of therapy, days	0.090	0.037	6.053	1.095	1.019–1.176	0.014^*a*^
Albumin, g/L	0.096	0.027	12.280	1.101	1.043–1.161	0.000^*a*^

^*a*^ *P* < 0.05.

^*b*^ Dependent variable: the effectiveness of ganciclovir treatment was determined by CMV negativization or increased circulating threshold (CT) (value = 1), while decreased CMV circulating threshold post-treatment indicated ineffectiveness (value = 0).

### Effect of renal function and RRT on plasma drug concentration

**Fig 1:**
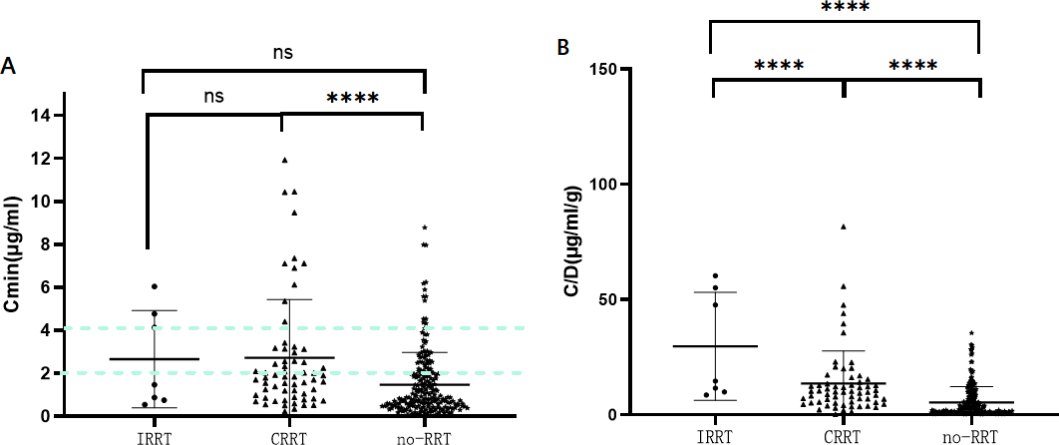
Effects of renal replacement therapy on Cmin (**A**) and C/D (**B**). Note: ****P < 0.001; ns, not significant (*P* > 0.05); trough concentration (Cmin), standard concentration adjusted by ganciclovir daily dose (C/D). The dashed green line indicates the concentration range of the target valley.

In this study, 281 plasma concentrations were recorded, including 62 cases (22.06%) with CRRT and seven cases (2.49%) with IRRT. Data were divided into CRRT, IRRT, and no renal replacement therapy (no-RRT) groups. The Cmin was significantly higher in the CRRT group (2.72 ± 2.71 µg/mL) compared to the no-RRT group (1.47 ± 1.51 µg/mL, *P* < 0.0001) and slightly higher than the IRRT group (2.65 ± 2.27 µg/mL), though not statistically significant (*P* = 0.995). For the concentration adjusted by the tacrolimus daily dose (C/D), the mean value was highest in the IRRT group (29.76 ± 23.46 µg/mL/g) and lowest in the no-RRT group (5.46 ± 6.80 µg/mL/g), with significant differences among these groups (*P* < 0.0001) (Fig. 1).

**Fig 2:**
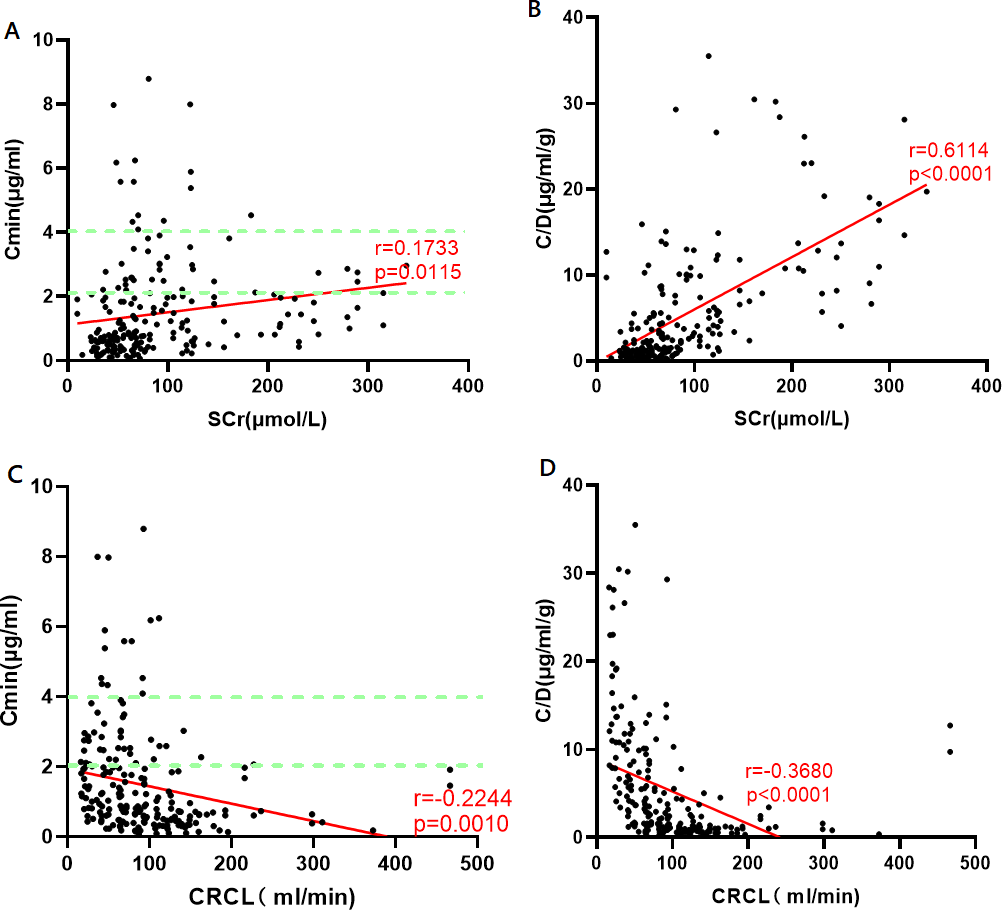
Effects of SCr on Cmin (**A**) and C/D (**B**) in the absence of renal replacement therapy. Effects of CRCL on Cmin (**C**) and C/D (**D**). The red line denotes relevant trends, while the dashed green line shows the target valley’s concentration range. ****P < 0.001; trough concentration (Cmin), standard concentration adjusted by the daily ganciclovir dose (C/D), serum creatinine (SCr), and creatinine clearance (CRCL)

We examined renal function’s impact on Cmin in patients not undergoing RRT. Using 212 plasma concentrations, we employed SCr and CRCL (Cockcroft-Gault equation) as renal function indices and assessed their effect on Cmin and C/D, creating scatter plots. Spearman’s correlation analysis revealed a significant positive correlation between SCr and both Cmin (*r* = 0.1733, *P* = 0.0115) and C/D (*r* = 0.6114, *P* < 0.0001). Conversely, CRCL showed a significant negative correlation with Cmin (*r* = −0.2244, *P* = 0.0010) and C/D (*r* = −0.3680, *P* < 0.0001). Refer to Fig. 2 for details.

### Analysis of independent factors influencing ganciclovir Cmin

Further multiple linear regression analyses of ganciclovir Cmin indicated correlations with disease type, HGB, SCr, and CRRT. Diabetic patients exhibited a 0.869 µg/mL decrease in ganciclovir Cmin compared to non-diabetics (*P* = 0.001). Transplant patients had a 0.842 µg/mL reduction in Cmin compared to non-transplant patients (*P* = 0.008). Each 1 g/L increase in the HGB level resulted in a 0.011 µg/mL decrease in Cmin (*P* = 0.034). Additionally, each 1 µmol/L increase in SCr corresponded to a 0.006 µg/mL increase in Cmin (*P* = 0.000). The administration of CRRT increased ganciclovir Cmin by 0.967 µg/mL (*P* = 0.000). The predictors of ganciclovir Cmin are detailed in Table 5.

**TABLE 5:** Independent influencing factors of Cmin^*a*^^,^^*b*^

Parameters	Estimate coefficients	Std. error	*t*	VIF	*P*
(Intercept)	2.188	0.497	4.403		0.000
Diabetes	−0.869	0.263	−3.300	1.023	0.001
Transplantation	−0.842	0.315	−2.672	1.070	0.008
Hemoglobin, g/L	−0.011	0.005	−2.133	1.023	0.034
SCr, μmol/L	0.006	0.001	4.541	1.108	0.000
CRRT	0.967	0.263	3.679	1.032	0.000
*F*		11.600			
*R* ^2^		0.174			
Adjusted *R*^2^		0.159			
*P*		0.000			

^*a*^ Stepwise multiple linear regression was conducted with inclusion and exclusion criteria set at 0.05 and 0.10, respectively (*N* = 281). Scr denotes serum creatinine, and CRRT stands for continuous renal replacement therapy.

^*b*^ Note: serum creatinine (SCr), continuous renal replacement therapy (CRRT), variance inflation factor (VIF).

The final multiple linear regression equation is as follows:

Cmin = 2.188 − 0.869 × DM − 0.842 × TP − 0.011 × HGB + 0.006 × SCr + 0.967 × CRRT

where, Cmin is the trough concentration. “DM = 1” if the patient has diabetes; otherwise, “DM = 0” ; “TP = 1” if the patient has transplantation; otherwise, “TP = 0” ; and “CRRT = 1” if the patient has CRRT; otherwise, “CRRT = 0.”

### Ganciclovir TDM and hemocytopenia

**Fig 3:**
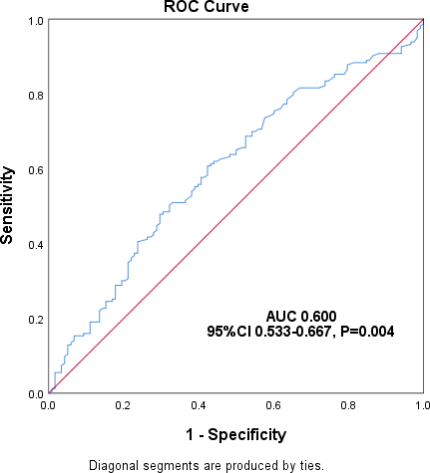
ROC curve analysis to predict the risk of ganciclovir concentration-related hemoglobin reduction occurrence.

Leukopenia, neutropenia, thrombocytopenia, and HGB reduction incidence rates were 59.73, 66.44, 57.72, and 63.76%, respectively, as detailed in Table 6. A significant correlation existed between ganciclovir Cmin and HGB reduction (*r* = 0.139, *P* = 0.020). The ROC curve analysis (Fig. 3) indicated a threshold ganciclovir Cmin value of 0.985 µg/mL, with an AUC of 0.600 (95% CI, 0.533–0.667) (*P* = 0.004). No significant correlation was found between ganciclovir Cmin and reductions in other blood cell counts.

**TABLE 6:** Statistics of related adverse reactions (*n* = 149)

Toxicity	Cases (%)
Leukopenia	89 (59.73）
White blood cell count＜3 × 10^9^/L	12 (8.05）
Decrease in white blood cell count from baseline＞20%	75 (50.34）
Neutropenia	99 (66.44）
Neutrophil count＜1.5 × 10^9^/L	4 (2.68）
Decrease in neutrophil count from baseline＞20%	82 (55.03）
Thrombocytopenia	86 (57.72）
Blood platelet count＜100 × 10^9^/L	34 (22.82）
Decrease in platelet count from baseline＞50%	20 (13.42）
Decreased hemoglobin	95 (63.76)
Hemoglobin <80 g/L	53 (35.57)
Decrease in hemoglobin from baseline >20%	30 (20.13)

### Ganciclovir TDM and hepatic or renal adverse effects

**Fig 4:**
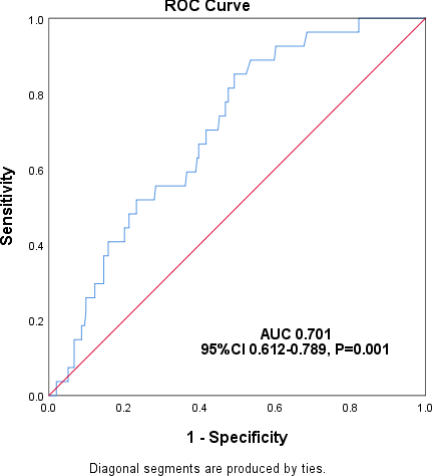
ROC curve analysis to predict the risk of ganciclovir concentration-related blood creatinine elevation occurrence.

No significant link was found between ganciclovir levels and liver function adverse reactions (ALT, AST, and TBIL), while a notable correlation existed between ganciclovir concentrations and elevated blood creatinine adverse reactions (*r* = 0.134, *P* = 0.025). The ROC curve analysis revealed that the threshold ganciclovir Cmin value for adverse reactions with increased SCr was 0.995 µg/mL, with an area under the curve of 0.701 (95% CI, 0.612–0.789) (*P* = 0.001)(Fig. 4).

## DISCUSSION

The optimal ganciclovir exposure in adults and children is undefined. Nokta et al. (23) showed that inhibition occurred at various concentrations. PK/PD studies have not confirmed whether ganciclovir is concentration- or time-dependent. Case studies (24–26) suggest TDM for optimizing ganciclovir regimens. Different centers have established specific target ranges for ganciclovir TDM (10, 20). In our study, only 19.22% of patients with confirmed CMV lung infections achieved the target Cmin range of 2.0–4.0 μg/mL, with 70.82% below 2.0 µg/mL. Despite the low Cmin attainment rate, our CMV negativization rate was 68.46%, and the overall treatment efficacy was 73.15%, indicating good clinical outcomes similar to other studies (10, 22, 27). The discrepancy between low Cmin attainment and high cure rates may be due to the pharmacological activity of the intracellular triphosphate form of ganciclovir (28), suggesting that current Cmin target ranges may be set too high.

CRRT increased ganciclovir Cmin, whereas IRRT had no significant effect. After dose adjustment, the highest concentrations were in intermittent dialysis patients. Galar et al. (22) found higher Cmin in hemodialysis patients without evaluating different modalities. Another study (20) in transplant patients showed lower ganciclovir Cmin in the IRRT group compared to the CRRT group, though not statistically significant. An earlier study (29) reported that a single 4 h dialysis session reduced plasma concentration by about 50%. Limited research on dialysis modalities suggests considering different hemodialysis durations when dosing ganciclovir. In patients not on renal replacement therapy, Cmin or C/D correlated significantly with renal function; a better renal function meant lower ganciclovir exposure. Ganciclovir elimination depends entirely on renal function, with evidence (20) showing sub-target Cmins in patients with eGFR > 90 mL/min/1.73 m². These findings stress the need for further studies to optimize ganciclovir dose adjustments for patients with renal insufficiency.

Besides blood creatinine and CRRT, our study revealed that diabetes mellitus, organ transplantation, and HGB levels at the time of TDM also independently influence ganciclovir Cmin. Contrasting with a prior study (22) that found low eGFR, but not solid organ transplantation, to significantly impact Cmin, our findings differ possibly due to the predominance of lung transplantation (21/29, 72.41%) in our study compared to heart (10/26, 38.46%) and liver (11/26, 42.31%) transplants in the previous study. The effects of solid organ transplantation on ganciclovir Cmin require further research. The impact of underlying diseases and blood cells on ganciclovir Cmin also necessitates additional investigation. Our results suggest that the varying pathophysiological states in patients underscore the utility of TDM in understanding *in vivo* ganciclovir exposure.

This study, along with findings by Gatti et al. (27) and Ritchie et al. (13), found no significant correlation between ganciclovir Cmin and its efficacy in CMV eradication or clinical improvement possibly due to the wide variations in IC50 (0.1 to 1.7 mg/L) and IC90 (up to 3.5 mg/L) required for CMV replication reduction (27). The binary logistic regression analysis revealed that prolonged ganciclovir use and higher patient albumin levels were favorable prognostic factors. A Dutch study (30) attributed the slow reduction in the CMV viral load with standard dosing to inadequate dosing or poor immune status, suggesting that enhancing the patient’s immune response and extending ganciclovir treatment under inadequate exposure conditions could promote viral clearance.

Ganciclovir TDM mitigates toxicity. Our study found a significant correlation between ganciclovir Cmin and HGB reduction, with a 0.985 µg/mL threshold for HGB reduction, a novel finding (20, 27). Clinical studies show varying results regarding ganciclovir Cmin and toxicity. Our findings necessitate further investigation. The adverse event thresholds were below our institution’s target range for ganciclovir Cmin (2.0 vs. 4.0 µg/mL), with low actual Cmin compliance rate (19.22%) and high therapeutic efficacy (73.15%), suggesting high Cmin may be nonessential. Additional research should determine if lowering Cmin ensures efficacy and safety.

Our study found that ganciclovir Cmin significantly influenced the incidence of adverse reactions linked to elevated blood creatinine. The ROC analysis determined a critical ganciclovir Cmin of 0.995 µg/mL associated with these adverse reactions. However, the link between ganciclovir and severe nephrotoxicity remains unclear, as no studies have confirmed a quantitative relationship between ganciclovir Cmin and nephrotoxicity occurrence. Only one study on pediatric hematopoietic cell transplant recipients (31) reported that intravenous ganciclovir was associated with severe renal dysfunction requiring renal replacement therapy.

Our study offers guidance on ganciclovir use and therapeutic drug monitoring (TDM) in clinical practice. Healthcare providers should not adjust doses for CMV pneumonia patients solely based on ganciclovir Cmin levels not meeting traditional targets. Instead, they should consider clinical symptoms, viral load changes, and other indicators comprehensively. We identified factors influencing ganciclovir Cmin, including diabetes, organ transplantation, hemoglobin and serum creatinine levels, and CRRT use. These factors can help clinicians adjust doses more precisely. For example, diabetes or transplant patients may need higher doses, while those with renal insufficiency or on CRRT should have doses carefully adjusted to avoid toxicity. We also determined Cmin thresholds for predicting adverse reactions related to hemoglobin reduction and elevated serum creatinine. Healthcare providers can use these thresholds with patient-specific conditions to take preventive measures, such as adjusting doses, increasing monitoring frequency, and closely observing symptoms and lab indicators. Other factors like age, comorbidities, and concomitant medications should also be considered. By integrating these factors, clinicians can optimize treatment plans and reduce adverse events using TDM results.

The study has some limitations. Recent research indicates that AUC24h more accurately predicts clinical outcomes. Only ganciclovir Cmin was collected in our study without considering the AUC and peak concentrations because of retrospective data collection. Additionally, our study used the CT-based human cytomegalovirus nucleic acid assay, rather than the viral load in IU/mL as recommended by WHO guidelines.

### Conclusion

This study on ganciclovir therapeutic drug monitoring found no link between ganciclovir Cmin and clinical efficacy; however, Cmin below the target range did not impact efficacy outcomes in our patients. For the first time, we identified Cmin thresholds associated with a decrease in HGB and an increase in blood creatinine levels at 0.985 and 0.995 µg/mL, respectively. Renal replacement therapy was observed to likely elevate ganciclovir Cmin, indicating the necessity for dosage adjustments based on renal function and therapy. These results underscore the importance of ganciclovir use in TDM.

## Data Availability

The researchers confirm the accuracy of the data provided for the study, and they are available at https://pan.baidu.com/s/1LJSdd8opLWm5D6VYgaA0iQ.
